# Genomic prediction with machine learning in sugarcane, a complex highly polyploid clonally propagated crop with substantial non‐additive variation for key traits

**DOI:** 10.1002/tpg2.20390

**Published:** 2023-09-20

**Authors:** Chensong Chen, Owen Powell, Eric Dinglasan, Elizabeth M. Ross, Seema Yadav, Xianming Wei, Felicity Atkin, Emily Deomano, Ben J. Hayes

**Affiliations:** ^1^ Queensland Alliance for Agriculture and Food Innovation University of Queensland Queensland Australia; ^2^ Sugar Research Australia Mackay Australia; ^3^ Sugar Research Australia Gordonvale Australia; ^4^ Sugar Research Australia Indooroopilly Australia

## Abstract

Sugarcane has a complex, highly polyploid genome with multi‐species ancestry. Additive models for genomic prediction of clonal performance might not capture interactions between genes and alleles from different ploidies and ancestral species. As such, genomic prediction in sugarcane presents an interesting case for machine learning (ML) methods, which are purportedly able to deal with high levels of complexity in prediction. Here, we investigated deep learning (DL) neural networks, including multilayer networks (MLP) and convolution neural networks (CNN), and an ensemble machine learning approach, random forest (RF), for genomic prediction in sugarcane. The data set used was 2912 sugarcane clones, scored for 26,086 genome wide single nucleotide polymorphism markers, with final assessment trial data for total cane harvested (TCH), commercial cane sugar (CCS), and fiber content (Fiber). The clones in the latest trial (2017) were used as a validation set. We compared prediction accuracy of these methods to genomic best linear unbiased prediction (GBLUP) extended to include dominance and epistatic effects. The prediction accuracies from GBLUP models were up to 0.37 for TCH, 0.43 for CCS, and 0.48 for Fiber, while the optimized ML models had prediction accuracies of 0.35 for TCH, 0.38 for CCS, and 0.48 for Fiber. Both RF and DL neural network models have comparable predictive ability with the additive GBLUP model but are less accurate than the extended GBLUP model.

## Core Ideas

- Machine learning has been proposed to capture non‐additive effects in genomic prediction.
- We compare extended GBLUP models (extended to model dominance and epistasis) with several machine learning methods (including deep learning methods) for genomic prediction in sugarcane.
- Only optimized random forest was almost competitive with extended GBLUP for genomic predictions across traits.

**Abbreviations**
BLUP: best linear unbiased prediction
CCS: commercial cane sugar
CNN: convolutional neural network
DL: deep learning
FAT: final assessment trial
GBLUP: genomic best linear unbiased prediction
GEBV: genomic estimated breeding value
GS: genomic selection
ML: machine learning
MLP: multilayer perceptron
MSE: mean squared error
PVE: proportion of SNP variance explained by SNP effects
RF: random forest
RKHS: reproducing kernel Hilbert space
SNP: single nucleotide polymorphism
TCH: total cane harvested

## 1 INTRODUCTION

Genomic selection (GS) has been proposed as an approach to increase genetic gains in many breeding programs (Goddard & Hayes, 2007; Meuwissen et al., 2001). It overcame the drawback of marker‐assisted selection which was limited to markers with significant associations. In contrast, genomic selection uses single nucleotide polymorphism (SNP) markers across the whole genome to increase the likelihood of the markers being in linkage disequilibrium with quantitative trait loci of the target trait. GS has proven successful and has increased the genetic gain for many agricultural species via shorter breeding cycles and higher prediction accuracy (Jonas & de Koning, 2013). Although GS has been widely implemented in diploid genomes and resulted in substantial production improvements, for polyploid species GS is still in its early validation stage despite its potential advantages (Beyene et al., 2021; Juliana et al., 2020; Krishnappa et al., 2021; Lozada & Carter, 2020; Xu et al., 2021).

The major challenge in genomic prediction is the “small n big p problem”: estimating the effects of a large number of markers (*p*) using phenotype information from a relatively smaller number of individuals (*n*) (Meuwissen et al., 2001). Linear mixed models have been widely used for genomic prediction including SNP best linear unbiased prediction (SNP‐BLUP) and genomic best linear unbiased prediction (GBLUP), which assume normal distributions of marker effects and shrink the effects accordingly. These models have been shown to be reliable and robust in a range of studies (Meuwissen et al., 2001; Misztal & Legarra, 2017; VanRaden, 2008; Zhang et al., 2021). Bayesian approaches for genomic prediction offer more flexibility in the choice of prior distributions of underlying genetic architectures of complex traits (Erbe et al., 2012; Habier et al., 2011; Meuwissen et al., 2001; Wolc & Dekkers, 2022). All the models assume additive gene action.

Efforts have been made to capture nonadditive genetic variation within linear mixed model frameworks via extensions of the standard additive GBLUP model to include dominance effects (Jannink et al., 2010; Su et al., 2012; Varona et al., 2018; Zhao et al., 2015) and epistatic effects (Vitezica et al., 2017; Vitezica et al., 2013; Wang et al., 2012). However, such extended approaches are computationally demanding and do not address nonadditivity beyond pairwise interactions. Kernel‐based approaches, such as the use of reproducing kernel Hilbert space regression (RKHS) (Berk, 2008), have been proposed to capture gene‐by‐gene (GxG) epistatic effects and genotype‐by‐environment (GxE) interactions more effectively (Cuevas et al., 2016; Morota & Gianola, 2014). The RKHS was shown to result in a slightly higher prediction accuracy for total cane harvested (TCH) than additive GBLUP but did not show significant improvements compared to extended GBLUP models (Yadav et al., 2021). To date, genomic prediction in sugarcane has used GBLUP, extended GBLUP, Bayesian alphabets, and kernel algorithms (Hayes et al., 2021; Islam et al., 2021; Voss‐Fels et al., 2021; Yadav et al., 2021). However, predicting performance in sugarcane is still challenging because of its high level of polyploidy and interspecific origins (Garsmeur et al., 2018; Piperidis et al., 2010; Thirugnanasambandam et al., 2018).

With the recent advancements in computational power and increasing availability of genomic data, machine learning (ML) algorithms have become more accessible and easier to implement. Machine learning approaches are interesting to consider for species with highly complex genomes, as they are purported to be capable of capturing nonadditive and interaction effects (Azodi et al., 2019; Costa‐Neto et al., 2021; Pérez‐Enciso & Zingaretti, 2019).

**Core Ideas**

- Machine learning has been proposed to capture non‐additive effects in genomic prediction.
- We compare extended GBLUP models (extended to model dominance and epistasis) with several machine learning methods (including deep learning methods) for genomic prediction in sugarcane.
- Only optimized random forest was almost competitive with extended GBLUP for genomic predictions across traits.

The first application of a neural network for genomic prediction was presented in 2011 to predict body mass index in mice using one hidden layer (Okut et al., 2011). Since then, more complex DL architectures have been used for genomic prediction including radial basis function neural networks, probabilistic neural network classifiers, and convolutional neural networks (CNNs) (Abdollahi‐Arpanahi et al., 2020; Crossa et al., 2019; Jubair et al., 2021; Montesinos‐López et al., 2018; Sandhu et al., 2021; Wilson & Izmailov, 2020; Zeng et al., 2021; Zingaretti et al., 2020).

Previous studies that have extended the conventional genomic prediction method of GBLUP, to capture nonadditive trait variation, have also demonstrated increases in prediction accuracy across different species (Bajgain et al., 2020; Ishimori et al., 2020; Yadav et al., 2021). Thus, the main objective of this study was to compare ML approaches, standard GBLUP, and extended GBLUP models for prediction accuracy of three traits in sugarcane, including TCH, commercial cane sugar (CCS), and fiber content (Fiber), covering a range of underlying genetic architectures. For this study, we used widely adopted ML models: random forest (RF, as a reference ML approach), multi‐layer perceptron MLP, and CNNs for genomic prediction of clonal performance for three economically important traits and benchmarked these methods against GBLUP and extended GBLUP. Specifically, the objectives of this study were to: (1) identify underlying factors contributing to differences in prediction accuracy across genomic prediction models, including the discovery of functional similarity between linear mixed model and ML genomic prediction models; and (2) investigate improvements in the prediction accuracy of ML methods via model optimization.

## 2 MATERIALS AND METHODS

### 2.1 Sugarcane genotyping

A total of 3006 sugarcane clones were genotyped using the 44K SC Affymetrix Axiom SNP array (Aitken et al., 2016). This array consisted of 58,028 single‐dose polymorphic markers, developed particularly for Australia and Brazil sugarcane breeding programs. When the array was developed, SNP markers following a diploid inheritance pattern were prioritized. Details of the SNP array are provided in Aitken et al. (2016).

Multiallelic markers were called as pseudo diploid genotypes from the array data. All heterozygous genotypes were measured as one single‐class genotype, from a pseudo‐diploid model during the genotype calling process. The quality control threshold for the SNP marker data was set as Affymetrix QC score of 0.6, minor allele frequency as 0.01, and 90% genotype calling rate, done using the R‐package “SelectionTools” version 19.3. 26,086 markers passed quality control step from the raw 44K markers. A very small number of missing genotypes were replaced by sampling 0, 1, or 2, according to the most likely genotype given the minor allele frequency for each SNP. The SNP data was coded as “0” for homozygous reference alleles, “2” for homozygous alternate alleles, and “1” for heterozygous genotypes.

### 2.2 Sugarcane phenotyping

Phenotypes for the 2912 clones used in this study were taken from final assessment trials (FATs) of Sugar Research Australia's breeding programs. The initial population comprised 25,000 genotypes from 250 bi‐parental crosses. FATs were conducted across four growing regions: “Northern,” “Burdekin,” “Central,” and “Southern” in Australia with four trials per region and year 2013 to 2017. For each region, clones were tested in FATs in up to five sites per year or series from 2013 to 2017. Each site had 108 to 330 clones planted in a 4 rows x 10 m plot following a p‐rep design. Only the plants from the middle two rows were used for data collection to avoid competition effects. Each series was harvested over 3 years as a plant crop, the first ratoon crop, and the second ratoon crop. Overall, there was an average replication rate of 22% within trials.

At each harvest, three main agronomical traits were measured: tons of cane per hectare (TCH), CCS, and Fiber (Table 1). The data for TCH were collected from the middle two‐row plots, and CCS and Fiber were mainly measured by SpectraCane (Berding & Marston, 2010) or press method (Queensland Bureau of Sugar Experiment Stations, 1984) with six randomly selected stalks. The industry partner supplying data for the project fitted a BLUP model accounting for spatial effects and trial effects (row and column fitted as fixed effects, trial within a region) in their routine evaluations. In detail, the genomic predictions were performed through two steps. First, to account for spatial variation in each trial and GxE interaction between trials and crops, a linear mixed model was fitted by considering the spatial and GxE effects to estimate BLUPs for each crop within a year‐crop using the BLUP methodology as the linear mixed model:

(1) $$Y\;=\;X\tau +Z_{g}u_{g}+Z_{p}u_{p}+\varepsilon$$

where **X** is the design matrix, τ is the vector of fixed effects as described above, $Z_{g}$ is the designed matrix, u_g_ is the vector of random genotype (clone) effects by trials harvests, u_p_ presents peripheral random effects associated with designed matrix $Z_{p}$, and the ε is the residual. Details are also described in Smith et al. (2007). Importantly, no pedigree information was incorporated in this stage of the analysis, and the BLUPs are not shrunk toward the pedigree. Then, we performed an additional fixed effect model that averaged between region, planting years, trails, and ratoon crops. The estimates of clones from this model were then used as phenotypes in ML and GBLUP strategies. It would be preferable at this level to fit these fixed effects simultaneously with clone effects in these models. However, for the ML models, this was not currently possible with the available software that is widely used to implement these models.

**TABLE 1:** Sugarcane clones shared within regions.

Region	Northern	Southern	Burdekin	Central
Northern	338	124	136	114
Southern	124	230	182	193
Burdekin	136	182	1049	137
Central	114	193	137	280
Total	712	729	1504	724

### 2.3 Genomic best linear unbiased prediction

Variance components were estimated using the genomic restricted maximum likelihood. Additive GBLUP and extended GBLUP models were fitted; the latter included dominance and epistatic random effects as well as additive effects used in the study (Equation [2]).

(2) Y=u+Za+Zd+Ze+ε,

(3) $$A_{\mathrm{jk}}=\;\frac{1}{N}\sum_{i\;=\;1}^{N}\frac{\left(x_{\mathrm{ij}}-2p_{i}\right)\left(x_{\mathrm{ik}}-2p_{i}\right)}{2p_{i}\left(1-p_{i}\right)},$$

(4) $$D_{\mathrm{jk}}=\;\frac{1}{N}\sum_{i\;=\;1}^{N}\frac{\left(x_{\mathrm{ij}}-2p_{i}^{2}\right)\left(x_{\mathrm{ik}}-2p_{i}^{2}\right)}{2p_{i}^{2}\left(1-p_{i}^{2}\right)},$$

where **y** is a vector of adjusted phenotypes, u is the mean, **Z** is the design matrix mapping phenotypes to individuals, **a** is a vector of additive effects, **d** is a vector of dominance effects, and **e** is a vector of epistatic effects for clone. Then, $a∼N\left(0,E\sigma_{a}^{2}\right),\;d∼N\left(0,D\sigma_{d}^{2}\right),\;e∼N\left(0,E\sigma_{e}^{2}\right)$, where $\sigma_{a}^{2},\;\sigma_{d}^{2},\;\sigma_{e}^{2}\;$are the additive, dominance, and epistatic variance, respectively. The additive (**A**) (Yang et al., 2011) and dominance (**D**) (Zhu et al., 2015) genomic relationship matrix was constructed with Equations (3) and (4), respectively, via the program “GCTA,” version 1.94 (Yang et al., 2011). The epistatic (**E**) relationship matrix was calculated by taking the Hadamard product of the additive relationship matrix (E=A∘A) (Cockerham, 1954; Jiang & Reif, 2020). ε is a vector of random residuals. The additive GBLUP (A) and extended GBLUP models (AD and ADE) were fitted with the program MTG2, version 2.22 (Lee & van der Werf, 2016). All methods were compared for their predictive ability for clonal performance of three traits: TCH, CCS, and Fiber. The prediction from the additive GBLUP was termed as estimated breeding values, while predictions from the extended GBLUP were termed predicted clonal performance (Yadav et al., 2021).

### 2.4 Machine learning methods

#### 2.4.1 Random forest

The RF, as a type of ensemble method, is formed by hundreds of estimators and is named for its use of decision trees. Each decision tree inside the RF is independent and allocated by M clones with subset SNPs bootstrapped from 26,086 SNPs. The prediction of the RF is made by averaging the predictions from all the decision trees (Breiman, 2001; Mienye et al., 2019).

In this study, the package scikit‐learn (Version 1.0.2) in Python (Pedregosa et al., 2011) was used to perform RF modeling. We selected and assessed two parameters during the RF optimization: the number of decision tree leaves (SNP subset) and number of trees (estimators). The RF number of trees tested was [50, 100, 200, 500], and the number of decision tree leaves tested [10, 25, 35, 50, 100, 300, 500, 1000, 2000, 5000], resulting in a total of 24 sets of hypermeters being tested. Mean squared error (MSE) was used as criteria for RF modeling, with error defined as distance between predictions and training set phenotypes.

#### 2.4.2 Multilayer perceptron

The multilayer perceptron (MLP) is a standard form of DL models composed of a series of fully connected neural layers. Layers in a MLP could be separated into three structural components: input layer, hidden layer, and output layer. The input layer is the first neural layer that incorporates SNP information. The neurons in the hidden and output layers have three primary attributes: the activation function, trainable weight, and bias. Neurons in a fully connected layer would receive all the information from the previous layer. In this study, the exponential linear unit (ELU) was used as an activation function for the forward propagation process (Equation (5)) (Clevert et al., 2015):

(5) $$\mathrm{ELU}\;\left(x\right)=\;\left\{\begin{array}{c} x\;\mathrm{if}\;x>0\\ \alpha (e^{x}-1)\;\mathrm{if}\;x\leq 0 \end{array}\right.,$$

where x is the input signal; α is a hyperparameter that is usually set to a small positive value, such as 0.1. Forward propagation in a single neuron with weight (w), bias (b), and input (x), and assume the output of the neuron is $y_{out}$ is represented in Equation (6):

(6) $$Y_{\mathrm{out}}=f_{a}\;\left(\sum w_{i}x_{i}+b\right).$$

The activation function (*f*_a_) transforms the input value (x) from the previous layer. The forward propagation process continues by multiplying the inputs with the weights (w) and adding bias (b). The results of this calculation are passed through again the activation function, which is considered as input to the next layer in the network. MSE was calculated between predicted and observed clonal performance within the training set. During backward propagation, the error is backpropagated from the output layer to the input layer. This allows the neural weights and bias to be updated using the gradient descent optimization algorithm. To update the weights and biases during backpropagation, the partial derivative of the cost function (defined as MSE) with respect to the weights and biases is calculated to minimize the error between the model prediction and observation:

(7) $$\Delta w_{j}\;=\;-\alpha \frac{\partial E}{\partial w_{j}}\;=\;-\alpha \;\frac{\partial E}{\partial o_{j}}\frac{\partial o_{j}}{\partial \sum {\left(w_{i}x_{i}+b\right)}_{j}}\frac{\partial \sum {\left(w_{i}x_{i}+b\right)}_{j}}{\partial o_{j-1}},$$

(8) $$\Delta b_{j}\;=\;-\alpha \frac{\partial E}{\partial b_{j}}\;=\;-\alpha \;\frac{\partial E}{\partial o_{j}}\frac{\partial o_{j}}{\partial z_{j}},$$

where ΔwandΔb are the changes in weights and biases during the backward propagation, calculated based on estimated error E, scaled by learning rate α, and o is the output value of the layer (Equations (7) and (8)).

Two hyper‐parameters were optimized for the MLP: model depth (hidden fully connected layer numbers) (within options 4, 8, 16) and model width (number of neurons per hidden layer) (within options 5, 10, 15, 25, and 45). All DL neural network models were trained for a total of 50 epochs at a learning rate of 0.0001. Following testing, the hidden layer configuration with the most consistent performance was chosen for use in the subsequent CNN modeling phase.

#### 2.4.3 Convolutional neural networks

CNN is an extended perceptron with additional convolutional layers. The CNN model used in the study was modified from the original structure called “LeNet” (Lecun et al., 1998). The convolutional layer contains a convolutional kernel, which is a key component of forward propagation. This layer transforms input data using convolution, activation, pooling, and normalization operators. The convolutional operation involves moving the convolutional kernel across the input data and performing element‐wise multiplication between the kernel weights and the input data, followed by a summation (Equation (9)):

(9) $$y_{j}=b+\sum_{k=-p}^{p}w_{k}x_{j-k},$$

where $y_{j}$ is the convolution output of position *j* in a final output vector *y*, *b* is the bias for the channel, $w_{k}$ is the weight in kernel, $x_{j-k}$ is the input signal at (j−k) position in overall length *m*, the size of kernel is usually ensured as an odd kernel length, and *k* is the position inside the kernel. Subsequently, activation function ELU was used to introduce nonlinearity into the network. Following that, a max‐pooling layer was used to perform down‐sampling by selecting the maximum value from a small region of the feature map, called as pooling window (Equation (10)):

(10) $$y=\max\left(x^{1},x^{2},\text{…}x^{n}\right).$$

The convolutional kernel also defines a matrix of weights that can be updated by backward propagation to minimize the loss (Equation (11)). The loss function (L) was the error between the predicted and observed phenotypes. Finally, the performance of the model was evaluated on the validation set and different hyperparameters were adjusted.

(11) $$\frac{\partial L}{\partial w_{k}}=\sum_{j=0}^{m-1}\frac{\partial L}{\partial y_{j}}x_{k-j}.$$

**FIGURE 1:**
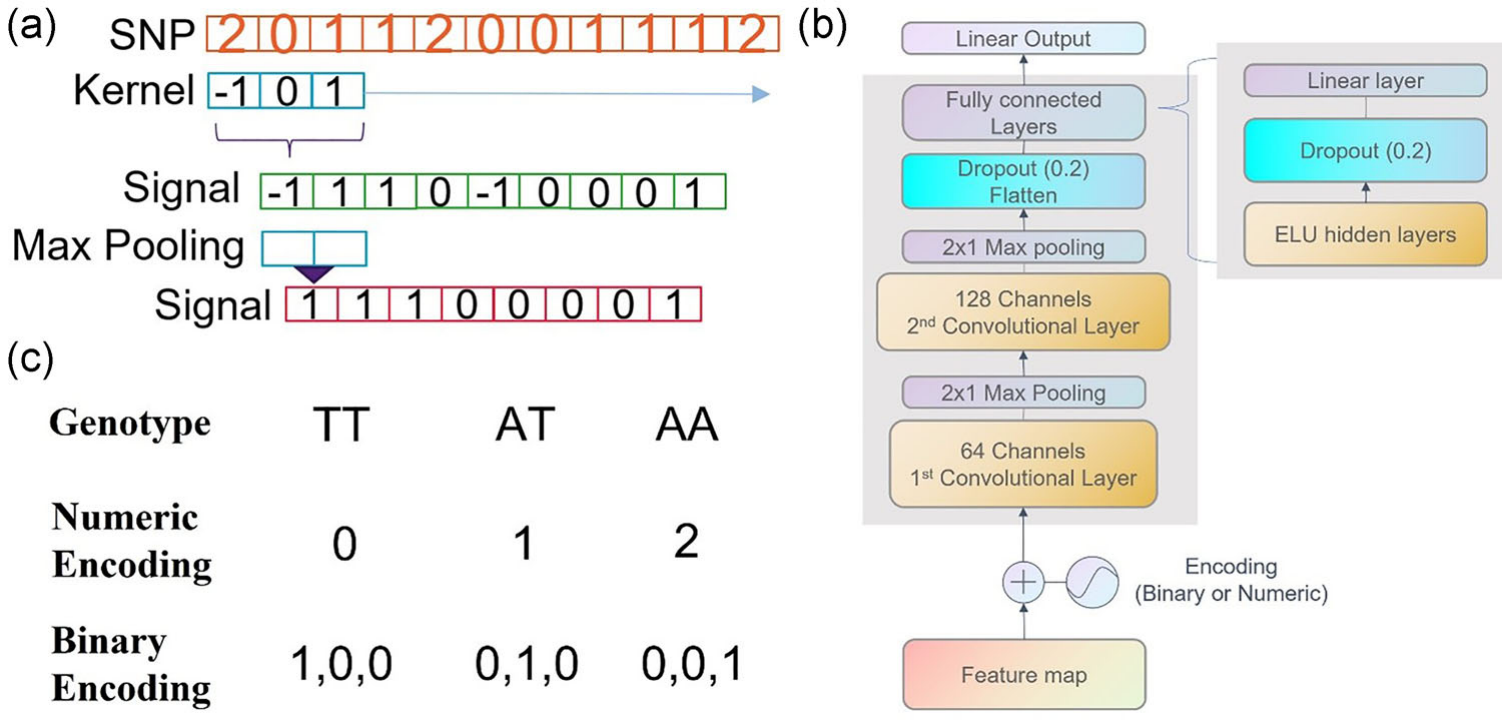
Structure of the convolutional neural network (CNN) used in this study. (a) An overview of the sequential convolutional layer and max‐pooling function in the CNN. (b) The CNN model contained two convolutional sections with different channels, followed by a fully connected perceptron, exponential linear unit (ELU) was selected as activation function for all the hidden layers, and the dropout rate was 20%. (c) Differences between single nucleotide polymorphism (SNP)‐encoding methods compared to genotypes.

In this study, our CNN structure had two convolutional layers. The first convolutional layer had a kernel length of five SNPs, a step length of three SNPs for moving the kernel forward, and generated 64 output channels. The second convolutional layer had a kernel length of three SNPs, a step length of three SNPs, and generated 128 output channels. All convolutional layers were followed by a max‐pooling layer that utilized a kernel length of two. A flattening step was used to combine signals in all channels to a single vector. The dropout step was included to avoid overfitting by randomly dropping neurons during each epoch. The dropout rate used was 0.2 (Figure 1).

Two SNP encoding methods, binary encoding and numeric encoding, were assessed in the CNN modeling (Figure 1c). The binary encoding treated the genotypes as categorical classes based on their actual genotypes (reference homozygous, heterozygous, and variant homozygous), and each label was represented using an independent binary panel. The numeric encoding, however, considered the genotyping data as numeric allele values (0, 1, 2).

All the DL neural networks including MLP and CNN models were run using the Python packages TensorFlow (Version 2.2.0) (Abadi et al., 2016) and Keras (Version 2.6.0) (Chollet, 2015).

#### 2.4.4 Optimization of hyper‐parameters for machine learning methods

Optimization of ML hyper‐parameters for each ML method was done by 10 repeats of train‐test‐validation with random initialization. The validation data was entirely excluded during the modeling. MSE was selected as the modeling criteria and loss function for all ML algorithms. Pearson's correlation between the phenotypes from the FATs and the predicted values in the validation set were selected as criteria for comparing among all models.

### 2.5 Comparison of predictive ability

To assess the accuracy of clonal prediction and the effect of training set size on method performance, we split the data into two training sets: training set that included the 2013–2015 FATs (1693 unique clones), and training set that included the 2013–2016 FATs (2221 unique clones). The validation set (clones from 2017) contained 691 unique clones. There were no clones that overlapped between the training and validation sets.

Prediction accuracy was measured as the Pearson's correlation between predictions and the adjusted phenotypes from the combined analysis. In total, we compared seven models including three GBLUP models (A, AD, ADE), two CNNs with different encoding methods (Numeric CNN, Binary CNN), and the MLP and the RF for evaluation of prediction accuracy. The standard error for the Pearson correlations was calculated as: $\surd \frac{1-r^{2}}{N-2}$, where N is the validation set size (Bowley, 1928).

### 2.6 Comparison of SNP contributions from RF and GBLUP

We used the RF and GBLUP models to compare SNP contribution. The proportion of variance explained by SNP effects (PVE) extracted from GBLUP model can be described as a measure of the importance of each SNP in predicting the phenotype and can be used to identify the most informative SNPs for a particular trait in RF model. The PVE was calculated using an extended GBLUP (AD) model that only included additive and dominance effects because comparing SNP importance for epistatic effects on an additive‐by‐ additive interaction basis was quite challenging.

The SNP effects in the GBLUP model were estimated using back solving (Yang et al., 2011), and the PVE was calculated based on these SNP effects, with modified equations described in Shim et al. (2015). The impurity‐based feature importance was calculated using the decrease of impurity formula (Equation (12)) (Nembrini et al., 2018; Scornet, 2020):

(12) $${\hat{\Gamma }}_{t}=\sum_{j=1}^{J}\hat{\phi_{j}}\left(t\right)\left(1-\;\hat{\phi_{j}}\left(t\right)\right),$$

where the $\hat{\phi_{j}}\left(t\right)$ represented the frequency of genotypes j in node t. The decrease of impurity is the difference between a node impurity and the weighted sum of the impurity measures of the child node (assume having two). The SNP contribution was then calculated as the SNP total PVE divided by the total PVE of all SNPs, across both additive and dominance components, and for RF model, the SNP contribution was calculated by SNP importance dividing sum of SNP importance. It could provide a measurement of the relative contribution of each SNP. The SNP PVE were partitioned by the genetic components (additive and dominance) and compared to SNP importance (impurity‐based feature importance) exported from the RF model in a similar scale. The SNP contribution should reflect the prior assumption of GBLUP models that all the SNP effects divided by genetic component follow a norm‐like distribution. Detailed equations for calculating PVE and SNP contribution are provided in Supporting Information.

## 3 RESULTS

### 3.1 GBLUP models

The broad‐sense heritability (H^2^) (estimated as $V_{g}/\left(V_{p}-\;V_{e}/r\right)$, where $V_{g},\;V_{e}\;,\mathrm{and}\;r$ were the genetic variance, error variance, and clone duplicates) was high for all traits. The average H^2^ is recorded as 0.72, 0.79, and 0.89 for TCH, CCS, and Fiber, respectively (Table 2).

**TABLE 2:** Phenotype summary and board‐sense heritability (*n* = 2912).

	TCH	CCS	Fiber
Min	−47.34	−5.66	−4.37
Mean	−6.74	0.2736	−0.71
Max	26.42	2.0913	5.71
*H* ^2^	0.72	0.79	0.89
SE	0.03	0.0296	0.03

Abbreviations: CCS, commercial cane sugar; Fiber, fiber content; TCH, total cane harvested.

**FIGURE 2:**
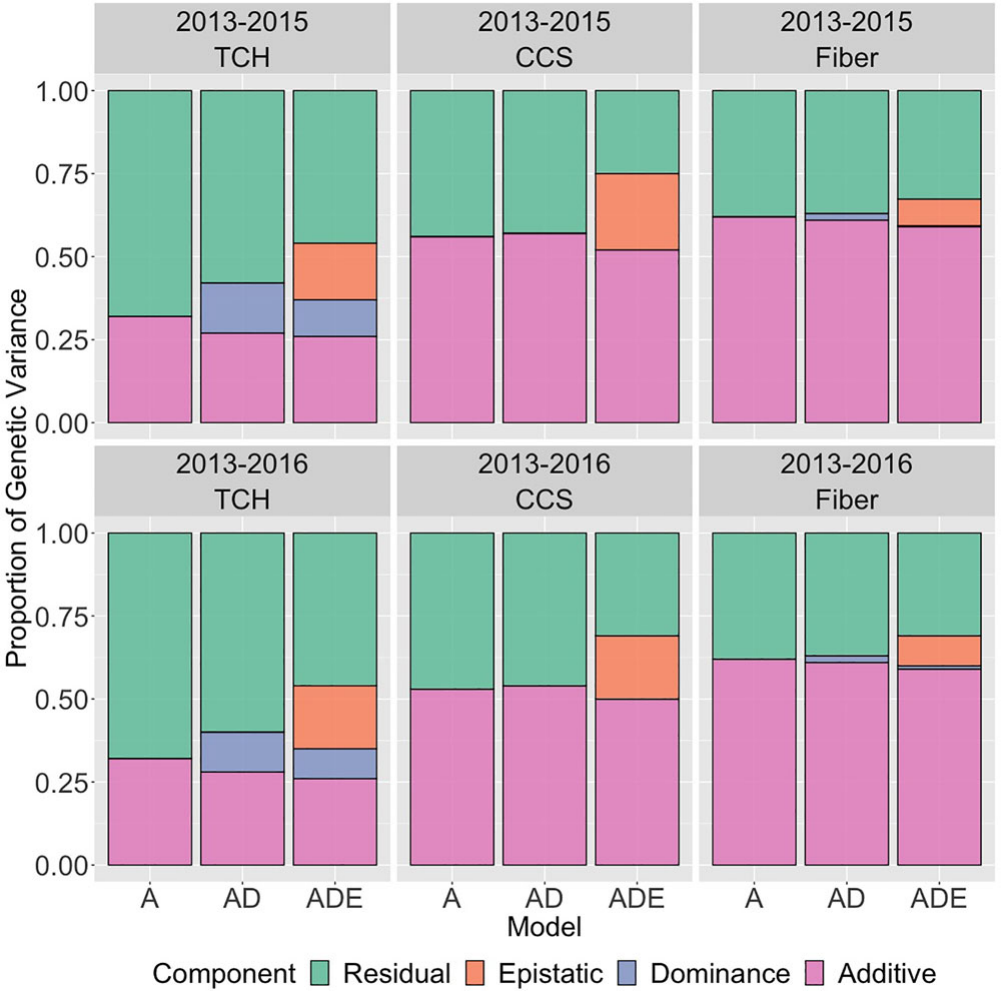
Proportions of genetic variances including additive, dominance, epistatic, and residuals estimated by genomic best linear unbiased prediction (GBLUP) models (A, AD, and ADE). Subplots were separated by traits and training sets. CCS, commercial cane sugar; Fiber, fiber content; TCH, total cane harvested.

For all traits, the proportion of additive effects was larger than the dominance and epistatic effects (Figure 2). TCH contained the highest ratio of dominance effects compared to CCS and Fiber.

Compared to the additive GBLUP model (A), the extended GBLUP models (AD, ADE) increased accuracy of predicting clonal performance for TCH by up to 0.05 for both training sets 2013–2015 and 2013–2016 (Table 3). No improvement for extended GBLUP over GBLUP was observed for either CCS or Fiber.

**TABLE 3:** Accuracy of predicting clonal performance for additive and extended genomic best linear unbiased prediction (GBLUP) models.

Model	Trait	R1^a^	R2^b^
A^c^	TCH	0.28	0.32
AD^d^		0.32	0.37
ADE^e^		0.33	0.37
A	CCS	0.39	0.43
AD		0.39	0.43
ADE		0.39	0.43
A	Fiber	0.45	0.48
AD		0.45	0.48
ADE		0.45	0.48

Abbreviations: CCS, commercial cane sugar; Fiber, fiber content; TCH, total cane harvested.

^a^ Model trained by 2013–2015 training set. Prediction accuracy measured by Pearson's correlation.

^b^ Model trained by 2013–2016 training set. Prediction accuracy measured by Pearson's correlation.

^c^ Additive GBLUP.

^d^ Additive dominance GBLUP.

^e^ Additive dominance epistatic GBLUP.

### 3.2 Comparison of performance from optimized methods in the validation set

**FIGURE 3:**
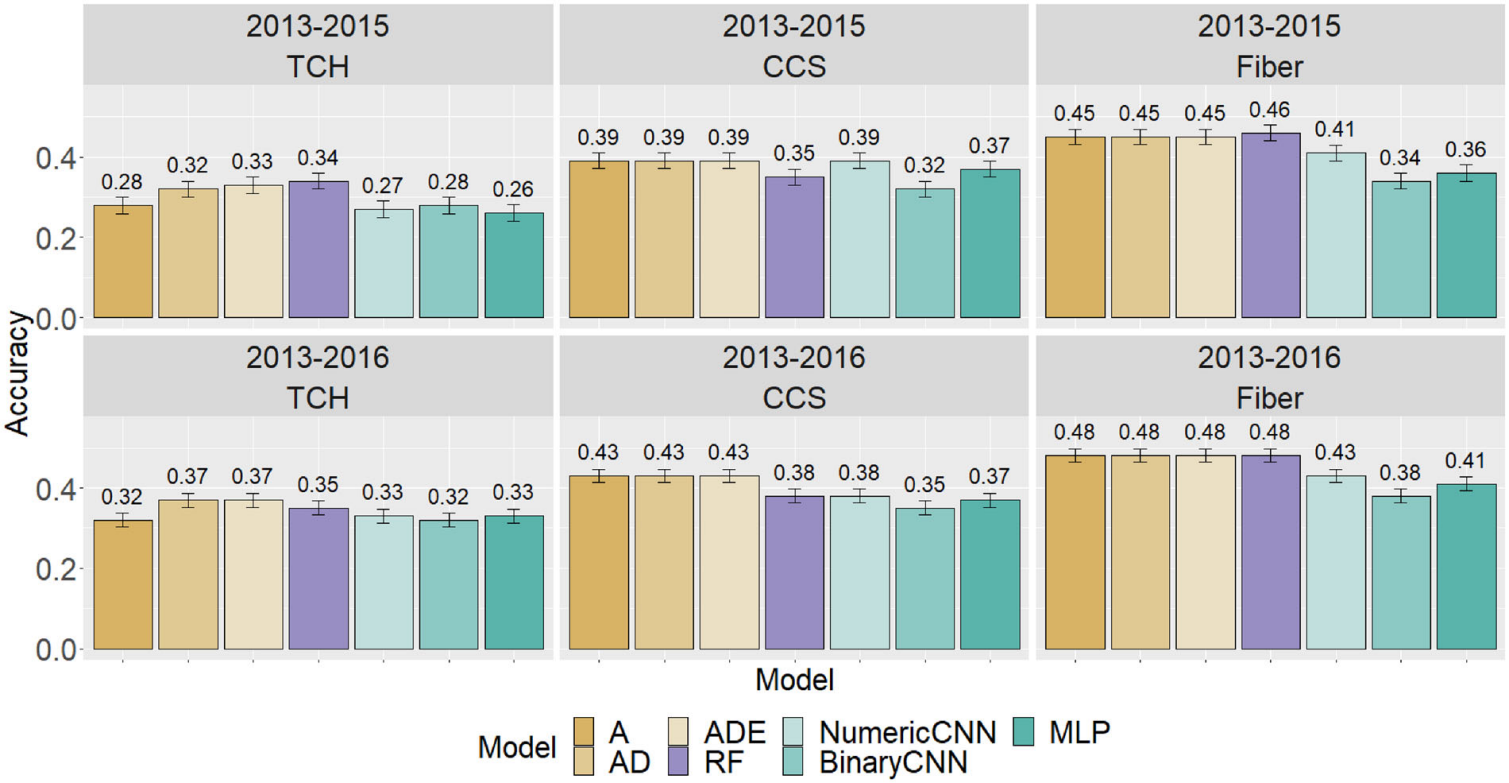
Comparison of accuracy of prediction from seven methods (GBLUP including A, AD, and ADE; RF, numeric CNN, binary CNN, and MLP) when the methods were used to predict clonal performance in the 2017 validation set. The hyper‐parameters in the ML methods were chosen based on performance from validations, divided by training sets including two scenarios (2013–2015 and 2013–2016). Accuracy was calculated as Pearson's correlation between predictions and observations. Error bars on the calculations were calculated as: $\surd \frac{1-r^{2}}{N-2}$. CCS, commercial cane sugar; CNN, convolutional neural network; Fiber, fiber content; GBLUP, genomic best linear unbiased prediction; MLP, multilayer perceptron; RF, random forest; TCH, total cane harvested.

There was a benefit brought by the large training set (2013–2016) for all traits and all methods (Figure 3).

In general, ML models had lower prediction accuracies than extended GBLUP, except for RF, which showed comparable results with GBLUP and extended GBLUP in predicting TCH and Fiber. For TCH prediction, while using a smaller training set (2013–2015), the RF model performed highest mean accuracy than MLP and CNN models, even higher than three GBLUP models, but was surpassed by extended GBLUP with large dataset (2013–2016). For CCS prediction, all the ML models performed less accurately than GBLUP models. Finally, for the prediction of Fiber, the RF model performed competitive with GBLUP models in both scenarios and was generally superior to CNN and MLP models.

### 3.3 Comparison of SNPs used for prediction in RF and GBLUP

**FIGURE 4:**
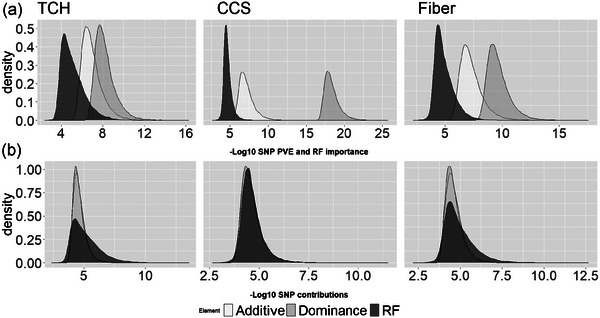
Analysis of SNP PVE and contributions explained by extended genomic best linear unbiased prediction (GBLUP) and random forest (RF). (a) Distribution of single nucleotide polymorphism (SNP) explained proportion of phenotypic variances (PVE) extracted from RF and GBLUP trained by 2013–2016 training set. Whereas *Y* axis in both subplots is the density, *X* axis in subplot (a) is the minus 10‐logged SNP PVE and SNP importance. (b) Distribution of SNP contributions between extended GBLUP and RF models. The SNP contribution was calculated as its PVE value divided by the sum of PVEs belonging to this genetic component (additive, dominant, or RF importance).

For all the three traits, the additive genetic effects had the highest mean PVEs when the distribution of SNP PVEs was examined (Figure 4a). Furthermore, the SNP importance extracted from the CCS RF model described a significantly smaller amount of variation compared to TCH and Fiber. We calculated the SNP contributions as the percentage for a SNP PVE belonging to its genetic component. We observed a very similar pattern of distribution between GBLUP and RF, in CCS. Conversely, a small percentage of SNPs were measured as relatively lower or higher SNP contributions to both TCH and Fiber compared to GBLUP models, which resulted in a skewed distribution (Figure 4b). A few SNP were labeled as zero importance in the RF models.

## 4 DISCUSSION

Across traits and species, no consistent advantage has been observed for genomic prediction accuracy from ML methods relative to GBLUP (Bayer et al., 2021; Heslot et al., 2012; Jubair et al., 2021; Li et al., 2018; Ogutu et al., 2011; Reinoso‐Peláez et al., 2022; Sirsat et al., 2022; Zingaretti et al., 2020). The performance of ML models depends on the suitable choice of hyperparameters, which is not trivial and requires considerable computational resources. Despite previous evidence, ML models without a well‐tuned set of hyperparameters could result in either underfitting or overfitting. Additionally, a set of well‐performing parameters obtained from one dataset may not perform well in another dataset. This might explain the lack of consistent advantage of using ML models over linear mixed models for genomic prediction. (Abdollahi‐Arpanahi et al., 2020; Montesinos‐López et al., 2021; Sandhu et al., 2021; Zingaretti et al., 2020).

In this study, we compared GBLUP and extended GBLUP models with our selected ML candidates for genomic prediction of clonal performance in sugarcane, a crop characterized by substantial nonadditive variation (Yadav et al., 2021). Overall, we found the extended GBLUP models would consistently outperform ML methods. Ubbens et al. (2021) came to a similar conclusion, and demonstrated this was because the predictive ability of DL methods arose from genomic relatedness.

Among ML methods, RF demonstrated superior predictive performance compared to CNN and MLP. Notably, RF's performance was comparable with extended GBLUP that accounts for nonadditive effects in predicting TCH and Fiber. Our results were consistent with previous studies, in which the performances of ML models (RF, MLP, and CNN) with similar architectures were not universally competitive with either BLUP or Bayesian models, in both crop (i.e., maize, wheat, rice, sorghum, and soybean) and animal (cattle dairy) studies (Azodi et al., 2019; Gianola et al., 2022; Montesinos‐López et al., 2018).

One possible explanation for this phenomenon is that the prior assumption made in BLUP models, that is, many mutations of small effect contribute to the variation in complex agricultural traits, is a useful model of complex trait variation. The GBLUP and extended GBLUP methods ensure accuracy by leveraging this assumption, which, in the case of BLUP methods, represents a robust and strong prior belief. ML methods have no such assumptions and may require more optimization (and more data) to correctly “learn” the genetic architecture correctly for complex traits, rather than either overfitting or sub fitting. Future advancements in machine learning methods may benefit from incorporating prior assumptions, such as in an integrated Bayesian machine learning approach. By integrating prior knowledge and assumptions into the model, it is possible to improve the performance and interpretability of machine learning algorithms, particularly in complex agricultural trait prediction. This integration of Bayesian principles can enhance the predictive accuracy and reliability of ML models (Wilson & Izmailov, 2020).

### 4.1 Effect of ML architecture on accuracy of genomic predictions

As our variance analysis accounted the varying proportions of genetic components within TCH, CCS, and Fiber, we investigated both ML models and GBLUP models to determine whether modifications to the models would result in improved prediction performance, particularly in the presence of different genetic architectures. First, we could conclude that the MLP complexity including depth and width would not generally enhance accuracy of predictions (Figure S1). Then, the improved fitting performance in either MSE or accuracy in CNN can be attributed to the utilization of convolutional layers, enabling them to capture and identify specific and important patterns from SNP arrays. The performance discrepancies observed among the different subtypes of CNN models were partially influenced by the distinct approaches to SNP signalization. The numeric CNN using allelic SNP markers performed significantly better than binary CNN relying on categorical genotypes through binary portals. It suggested that the allelic information could be more important than genotypic information for utilizing genomic prediction using DL approaches (Figures S2 and S3).

Second, RF models predicting TCH require more predictors (SNP) involved as leaves to form decision trees, to detect SNP by SNP relationships than CCS and Fiber, but TCH predictions were less affected, also relied less on the decision tree population (Figure S4). The above differences in either model structure for neural network models, or the optimization preference for RF models, might be associated with genetic architecture of these traits. For example, in the case of TCH, trait variation includes a significant proportion of nonadditive effects. Overall, this suggests that different ML models will be required for traits with different genetic architecture.

To compare quantitative SNP effects to SNP RF importance, we transformed back solved SNP effects from the extended GBLUP (with additive and dominance effects) to SNP PVEs (proportion of explained phenotypic variance), as in Montesinos‐López et al., 2022 and Shim et al., 2015. The results of our study indicated that RF identified similar SNP contributions for the prediction model to GBLUP, particularly for CCS, a trait primarily driven by additive effects. For the other two traits (TCH and Fiber), RF models shared a majority of SNP ranked by importance with extended GBLUP. It does need to be pointed out that a few SNPs had to be removed from the comparison due to the zero importance in RF (removed in the graph due to the minus infinite log10 value). The analysis provided some evidence that RF models captured similar effects to the GBLUP and extended GBLUP models.

### 4.2 Current limitations and future directions

Although the ML models offer potential applications for genomic prediction by discovering patterns in complex data, there are still concerns about their implementation. First, optimizing model structure, especially for DL models, seems specific for every application. Parameters such as batch size, the inclusion of residual connections, normalization layers, and learning rates do not have a standardized approach for optimization across different studies and datasets.

Our results lead to several insights regarding the potential benefits of ML algorithms for agricultural traits controlled by a significant proportion of nonadditive SNP effects. However, our results and insights are somewhat limited by the fact that we used “diploidized” genotypes in a highly polyploid species (as this is how the array was designed), and this may have led to an underestimate of the performance of ML methods, which reportedly can deal with high order nonlinear interactions (Aitken et al., 2016; Wu et al., 1992; de C Lara et al., 2019).

Machine learning frameworks, especially for deep learning models, often require additional parameterization and are highly dependent on factors like model structure, epochs, batch size, and GPU efficiency. The large number of parameters involved can increase computational time, ranging from minutes to hours, which can be a disadvantage when using machine learning methods compared to other approaches. In our study, our extended GBLUP required 10 min of CPU time using a high‐performance computing node with 12 CPU cores. For comparison, training a single DL modeling such as MLP and CNN would take around 2 min via HPC node with same CPU cores and NVIDIA Tesla V100 Accelerator Unit, totally around 25 min to finalize a 10‐repeat assessment. However, in practice, ML models in our study could also be trained using other GPUs, such as desktop NVIDIA GeForce RTX 2060, and when we tried that 8 min was required to fit a similar CNN model.

Finally, the interpretation of DL models remains challenging as extracting useful information such as SNP effects and interaction effects from reshaped layers and with various activation functions could be very complicated. The highly nonlinear nature of DL models makes it difficult to directly interpret the contribution of individual SNPs or understand the underlying biological mechanisms.

## 5 CONCLUSION

This study demonstrated the application of several ML methods, including MLP, CNN, and RF, for predicting clonal performance in sugarcane, a crop with a highly polyploid and complex genome, and with large nonadditive variation for some traits. Our findings show that even using pseudo diploid genotype markers as a SNP resource, the performance of RF models was almost competitive with selected three GBLUP models that included additive, dominance, and epistatic effects, at least for TCH and Fiber. DL models required more effort for model optimization; while RF methods were more robustly competitive and required simpler parameterization.

For the ML approaches, we found that the SNP encoding methods and model structures had a variable effect on prediction accuracy across the three traits in the study, which had different genetic architectures (e.g., different proportions of additive, dominance, and epistatic variance). This study provided some insights into the importance of these parameters for genomic prediction across different traits. Our study provides future directions for the development of ML‐based genomic prediction models in sugarcane and other crops with complex genomes.

## AUTHOR CONTRIBUTIONS

**Chensong Chen**: Formal analysis; investigation; methodology; software; validation; visualization; writing—original draft; writing—review and editing. **Owen Powell**: Conceptualization; investigation; supervision; writing—review and editing. **Eric Dinglasan**: Conceptualization; investigation; supervision; writing—review and editing. **Elizabeth M. Ross**: Conceptualization; investigation; supervision; writing—review and editing. **Seema Yadav**: Investigation; resources; writing—review and editing. **Xianming Wei**: Data curation; project administration; resources; writing—review and editing. **F** **elicity** **Atkin**: Data curation; project administration; resources; writing—review and editing. **Emily Deomano**: Data curation; project administration; resources; writing—review and editing. **Ben J. Hayes**: Conceptualization; funding acquisition; investigation; methodology; project administration; supervision; writing—review and editing.

## CONFLICT OF INTEREST STATEMENT

The authors declare no conflicts of interest.

## Supporting information

Supplemental Material

## Data Availability

The in‐house scripts, program, and manual for genomic prediction are available in the GitHub repository: https://github.com/CCS‐voidBird/GS_composer
