# Supplementary material for: Genomic prediction with machine learning in sugarcane, a complex highly polyploid clonally propagated crop with substantial non‐additive variation for key traits
Source: Plant Genome. 2023 Sep 20;16(4):e20390. doi: 10.1002/tpg2.20390 (PMC12806881; doi:10.1002/tpg2.20390)
Supplement: Supplementary file 1 [file TPG2-16-e20390-s001.docx]

SUPPLEMENTAL MATERIAL

Performance of neural network models

During MLP tuning, adjustments to network attributes, including model depth and width, had an impact on the accuracy of predicting clonal performance within the training set (as shown in Supplemental Figure 1). However, changes in prediction accuracies were minor. A linear model was used to estimate the trend of accuracy changes while adjusting model structures. For TCH, the prediction accuracy was reduced with growing hidden layers (P-value=0.00024), while the accuracy improved with larger model width (P-value=0.0036). The hyper-parameter tuning had no reflection on CCS prediction accuracy. For fiber prediction accuracy, an increase in model width impaired accuracy (P-value=0.00098).

While comparing SNP signalization methods, we found that the prediction accuracy was generally significantly higher in Numeric CNN than Binary CNN for all traits (Supplemental Figure 2). This section also compared the validation MSE for both MLP and CNN model, and it was found that CNN models generally had lower MSE than MLP models. For fiber content prediction, the numeric CNN had significantly lower MSE than the binary CNN. However, the MSE from TCH models showed larger variation than other traits (Supplemental Figure 3). Finally, it should be noted that CNNs and MLPs had the same depth and width at their fully connected layers, and the only difference between CNNs was the SNP encoding method used (Supplemental Figure 3).

Supplemental Figure 1. Multilayer perceptron prediction accuracy. a. Mean accuracy with different MLP depth (numbers of MLP hidden layers). b. Mean accuracy comparison with different MLP width (adjusting number of neurons per hidden layer). X axis related to the value of MLP attributes, Y axis showed the accuracy (Pearson’s correlation) in 2017 validation set.

Supplemental Figure 2 Comparison of CNN subtypes based on SNP signalizations. The X axis is CNN categories (Binary CNN or Numeric CNN), The Y axis is the Pearson’s correlations of the validation.

Supplemental Figure 3 Comparison of validation MSE among MLP, Numeric CNN and Binary CNN models. MSE were calculated for models with training set scenario 2013-2016, using the validation set 2017. Besides convolutional layers, all the models in this comparison contained 8 fully connected hidden layers.

Performance of random forest models

RF models with training set 2013-2015 had more significant tunning improvements than training with larger training set (2013-2016) (Supplemental Figure 4a). Furthermore, the potential for model tuning varied across traits, with the RF models predicting CCS and fiber content requiring a larger tree population to achieve maximum prediction accuracy compared to the TCH model.

The effect of decision tree leaves (SNP subset) on prediction accuracy was also evaluated while keeping the tree population fixed (Supplemental Figure 4b). A linear model was used to assess the overall effect of decision tree leaves. It was found that for all three traits, increasing the number of leaves in decision trees significantly improved prediction accuracy, although the TCH prediction accuracy was slightly less dependent on tree leaves compared to that of CCS and fiber content.

Supplemental Figure 4 Optimization for RF models a. Accuracy of prediction from RF with different tree populations (number of estimators). Star marks reflected to significant level of accuracy changes. Annotations above X axis were the significances of coefficients estimated by the formula: Accuracy ~ Tree population. Y axis presented predict accuracy calculated as Pearson’s correlation. b. Accuracy of prediction with RF decision tree leaves across the three traits. The RF tree population was set as 200, P-values above the X axis were related to the significant of coefficients for decision tree leaves. Y axis presented predict accuracy calculated as Pearson’s correlation.

Estimation of PVE and SNP weight

The PVE for GBLUP and extended GBLUP was extended from its original version (Shim et al., 2015), the effect size in the original formula was replaced by SNP effect:

$$PVE = \frac{2\hat{\beta}^{2}MAF(1 - MAF)}{\sum_{\hat{\beta} \in(\hat{\beta_{A}}, \hat{\beta}_{D})}^{\hat{\beta}} [2\hat{\beta}^{2}\mathrm{MAF}\left( 1 - MAF \right) + \left( \mathrm{se}\left( \hat{\beta} \right) \right)^{2}2N MAF\left( 1 - MAF \right)]}$$

Where $\hat{\beta}$ is the estimated SNP effect (additive $\hat{\beta_{A}}$or dominance $\hat{\beta_{B}}$); MAF is the Minor frequency alleles for the selected SNP and genetic effect (additive or dominance); N is the sample size.

The $\mathrm{se}\left( \beta\right)^{2} = \frac{\sigma^{2}}{2N MAF\left( 1 - MAF \right)}$ where $\sigma$ is the variance of second component (other genetic variants or residuals)

Formula of SNP weight of variance:

$$\mathrm{Var}\left( \hat{\beta} \right) = \frac{\mathrm{Abs}\left( \hat{\beta} \right)Var(g)}{\sum_{i = 1}^{M} \mathrm{Abs}\left( \hat{\beta_{i}} \right)Var(g_{i})}$$

$\hat{\beta}$ is the estimated SNP effect (additive $\hat{\beta_{A}}$or dominance $\hat{\beta_{B}}$), M is the size of SNPs, g is the allele distribution for $i^{\mathrm{th}}$ SNP.

References

Shim, H., Chasman, D. I., Smith, J. D., Mora, S., Ridker, P. M., Nickerson, D. A., Krauss, R. M., & Stephens, M. (2015). A multivariate genome-wide association analysis of 10 LDL subfractions, and their response to statin treatment, in 1868 Caucasians. *PLoS One, 10*(4), e0120758.
